# Initial characterization of drug resistant cancer stem cells isolated from primary brain tumors (astrocytoma) cell lines generated from Saudi patients

**DOI:** 10.1186/1471-2164-15-S2-P53

**Published:** 2014-04-02

**Authors:** Ishaq Khan, Mohammed Bangash, Saleh S Baeesa, Awatif Jamal, Hanadi Qashqari, Adel Abuzenadah, Mohammed H AlQahtani, Ghazi Damanhouri, Hans-Juergen Schulten, Deema Hussein, Adeel Chaudhary

**Affiliations:** 1 Center of Excellence in Genomic Medicine Research, King Abdulaziz University, Jeddah, KSA; 2 King Fahd Medical Research Centre, King Abdulaziz University, Jeddah, KSA; 3 Medical College, King Abdulaziz University, Jeddah, KSA

## Background

The Cancer Stem Cells (CSCs) hypothesis proposes that malignant brain tumours are organized into aberrant cell hierarchies where a subset of parent CSCs replicate asymmetrically and unlimitedly to produce differentiated daughter cell [1,2]. These parent CSCs are highly adaptive and resistant to the chemotherapeutic drugs [3]. Developing new drugs that target CSCs requires a comprehensive understanding of the pharmacogenomics behavior of these cells [4,5]. Such understanding requires reliable *in vitro* and *in vivo* models that represent the beneficial patients. This project is set out to: i) Establish a collection of astrocytoma cell lines generated from Saudi patients, ii) Select drug resistant brain tumour CSCs, iii) Characterize drug resistant brain tumour CSCs individually, and iv) Deduce common features for drug resistant brain tumour CSCs.

## Materials and methods

A range of methodologies will be applied including, tumour cell line derivation methods, the clonogenic selection assay, cytogenetics profiles, neuronal cancer stem cells characterisation assays and *in vivo* tumourgenic assays.

## Results

Optimisation of methods used to establish astrocytoma cell lines generated from Saudi patients was completed. To date two novel primary astrocytoma cell lines have been derived. Full clinical data related to the cell lines was retrieved. Currently, optimisation of the clonogenic selection assay and immunostaining for cancer stem cells markers are under progress.

## Conclusions

Successful retrieval of primary astrocytoma cell lines was possible to accomplish. Important clinical information data relevant to the cell lines were obtained providing clear identification reference. Further work is required for the characterization of the drug resistant cancer stem cell component within these cell lines. Once completed, this research will assist in understanding some of the molecular mechanisms relevant to drug resistance, especially for patients in the Kingdom of Saudi Arabia.

## References

[B1] Dirks PB Brain tumour stem cells: the undercurrents of human brain cancer and their relationship to neural stem cells Philosophical Transactions of the Royal Society B: Biological Sciences 2008 15 1489 139 152 10.1098/rstb.2006.2017 PMC2605491 17309866

[B2] Khoshnevisan A An overview of therapeutic approaches to brain tumor stem cells Medical journal of the Islamic Republic of Iran 2012 15 1 31 23483074 PMC3587894

[B3] Quezada C Garrido W Oyarzún C Fernández K 5′ ectonucleotidase mediates multipledrug resistance in glioblastoma multiforme cells Journal of cellular physiology 2013 15 3 602 608 22833450 10.1002/jcp.24168

[B4] Maugeri-Saccà M Vigneri P De Maria R Cancer stem cells and chemosensitivity Clinical Cancer Research 2011 15 15 4942 4947 21622723 10.1158/1078-0432.CCR-10-2538

[B5] Reya T Morrison SJ Clarke MF Weissman IL Stem cells, cancer, and cancer stem cells Nature 2001 15 6859 105 111 11689955 10.1038/35102167

